# Evidence-Based Practices for Children, Youth, and Young Adults with Autism: Third Generation Review

**DOI:** 10.1007/s10803-020-04844-2

**Published:** 2021-01-15

**Authors:** Kara Hume, Jessica R. Steinbrenner, Samuel L. Odom, Kristi L. Morin, Sallie W. Nowell, Brianne Tomaszewski, Susan Szendrey, Nancy S. McIntyre, Serife Yücesoy-Özkan, Melissa N. Savage

**Affiliations:** 1 School of Education, The University of North Carolina at Chapel Hill, CB 3500 Peabody Hall, Chapel Hill, NC 27599 USA; 2 Frank Porter Graham Child Development Institute, The University of North Carolina at Chapel Hill, Campus Box 8040, Chapel Hill, NC 27599-8040 USA; 3 Present Address: College of Education, Lehigh University, Iacocca Hall, 111 Research Drive, Bethlehem, PA 18015 USA; 4 Department of Allied Health Sciences, The University of North Carolina at Chapel Hill, Bondurant Hall, Campus Box 7120, Chapel Hill, NC 27599-7120 USA; 5 Present Address: College of Health Professionals and Sciences, University of Central Florida, 12805 Pegasus Drive, Orlando, FL 32816 USA; 6 Present Address: Department of Special Education, Anadolu Üniversitesi, Eğitim Fakültesi, Özel Eğitim Bölümü, Tepebaşı, Eskisehir, 26470 Turkey; 7 Present Address: College of Education, University of North Texas, 1300 W. Highland St., Denton, TX 76201 USA

**Keywords:** Evidence-based practice, Focused intervention, Autism spectrum disorder, Children and youth

## Abstract

This systematic review describes a set of practices that have evidence of positive effects with autistic children and youth. This is the third iteration of a review of the intervention literature (Odom et al. in J Autism Dev Disorders 40(4):425–436, 2010a; Prevent School Fail 54(4):275–282, 2010b; Wong et al. in https://autismpdc.fpg.unc.edu/sites/autismpdc.fpg.unc.edu/files/imce/documents/2014-EBP-Report.pdf; J Autism Dev Disorders 45(7):1951–1966, 2015), extending coverage to articles published between 1990 and 2017. A search initially yielded 31,779 articles, and the subsequent screening and evaluation process found 567 studies to include. Combined with the previous review, 972 articles were synthesized, from which the authors found 28 focused intervention practices that met the criteria for evidence-based practice (EBP). Former EBPs were recategorized and some manualized interventions were distinguished as meeting EBP criteria. The authors discuss implications for current practices and future research.

**Supplementary Information:**

The online version contains supplementary material available at 10.1007/s10803-020-04844-2.

Autism is currently one of the most visible and widely discussed human conditions. Its increased prevalence has brought it to the attention of society in the United States, with world-wide recognition (Lord et al. 2020). Much discussion surrounds the conceptualization of autism as a disability or as a set of unique skills that can be viewed as strengths (Urbanowicz et al. 2019). Although there is truth in both, there is also much verification that the life course for many individuals with autism, from infancy and into adulthood, is challenging for them and their families (Shattuck et al. 2018). In efforts to have a positive impact on this life trajectory, personnel in early intervention, schools, clinics, and other human service programs search for practices that could be most effective when working with children and youth with autism (Lai et al. 2020). The increased prevalence of autism (Maenner et al. 2020) has also intensified the demand for effective educational and therapeutic services, and intervention science is providing mounting evidence about practices that positively impact outcomes. The purpose of this study was to identify a set of focused intervention practices that have clear scientific evidence of positive effects with autistic^1^ children and youth (i.e., evidence-based practices). For this paper, we define scientific evidence as reports in a peer-reviewed journal of an experimental study of acceptable methodological quality that addresses the efficacy of a focused intervention practice.

The imperative for establishing and continuing to update information about evidence-based practices (EBPs) is because the knowledge about the demographics, key ability features, and intervention science related to autism continues to advance. At the same time, there is also a purveyance of interventions that have little or no evidence of effectiveness and yet are described as “cutting edge” (Siri and Lyons 2014). For example, Paynter et al. (2020) recently noted that the Autism Research website operated by the National Autism Society in the U.K. (http://www.researchautism.net/autism-interventions/alphabetic-list-interventions) catalogued over 1000 interventions, and many of which lacked evidence. Lack of evidence does not mean that interventions are ineffective, as studies may not yet have been conducted. However, current human services policies in the U.S. requires that intervention practices have research evidence of their effectiveness (e.g., Individuals with Disabilities Education Act, Medicaid waiver provisions, insurance coverage regulations).

The contemporary focus on EBPs for children and youth with autism can be tracked back to Cochrane’s (1972) proposition that health and medical services be based on empirical, scientific evidence of its efficacy. The movement to base health practices on scientific evidence gained further traction through Sackett’s and colleagues’ (1996) advocacy of evidence-based medicine. An important contribution of the evidence-based medicine movement, which Cochrane also suggested, was that such identification and verification of evidence-based practice *is just the first step*. The selection and application of such scientifically based practices depends on the skills and wisdom of the health care worker in selecting appropriate practices for the individual and applying them with fidelity. This multi-step process of blending information about scientifically identified, efficacious practices with practitioners’ knowledge and skill has been adopted in the evidence-based movements in education (Davies 1999; Odom et al. 2005), psychology (American Psychological Association 2006) and other human services (American Speech and Hearing Association 2004).

A conceptualization of the multi-step process for moving from applied intervention research studies to a practitioner’s use of the science in their work with individual autistic children or youth appears in Fig. 1. Conducting individual intervention research studies and publishing them in peer review journals are the initial steps. Systematic reviews that identify EBPs, such as the review reported in this article, are a central step in the process. Translation of the information generated by the systematic review of EBPs into user-friendly information and supporting the use of that information through professional development and implementation science strategies (e.g., coaching, leadership, etc.) are subsequent necessary steps. The latter step would build practitioner’s knowledge and skill in selecting and implementing practices (Guldberg 2017). Any broken links in this process chain reduces the probability that the knowledge generated by research studies will be utilized in practice.

**Fig. 1:**
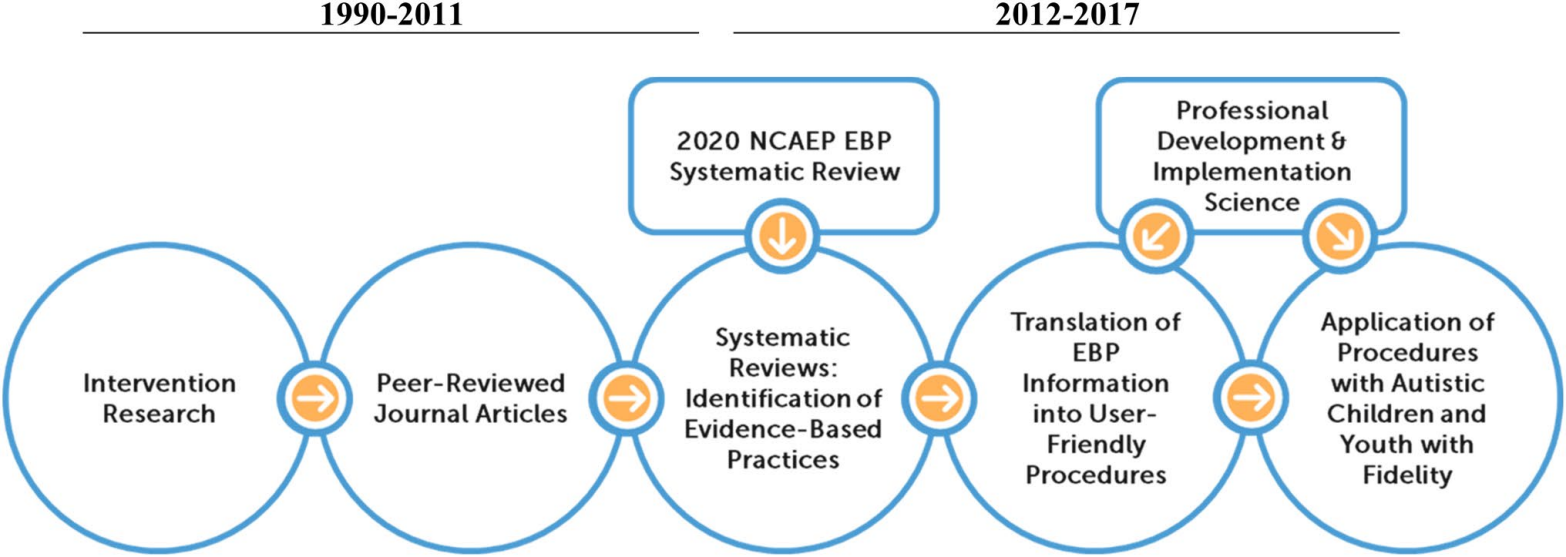
Research to practice process, with the current review reflecting the third step in the process

## Evidence-Based Intervention Approaches

Focused intervention practices and comprehensive program models (i.e., previously identified as comprehensive *treatment* models and modified because of the potentially ableist language implied) are two broad classes of interventions that appear in the research literature (Smith 2013). Focused intervention practices are designed to address a single skill or goal of a student with autism (Odom et al. 2010a, b). These practices are operationally defined and address specific learner outcomes. Teachers, clinicians, or other practitioners select and use the practices (e.g., prompting, reinforcement, time delay) in interventions or instruction that addresses a learners individual learning goal. Focused intervention practices could be considered the building blocks of educational programs for children and youth with autism.

In contrast, comprehensive program models consist of a set of practices designed to achieve a broad learning or developmental impact on the core deficits of autism (Odom et al. 2010a, b). Comprehensive programs are organized around a conceptual framework, procedurally manualized, focus on a breadth of outcomes, and are implemented with a substantial number of hours per week across one or more years (Odom et al. 2014). Examples of such programs are the early intensive behavior intervention program based on the UCLA Young Autism Project (Smith et al. 2000), the LEAP preschool model (Strain and Bovey 2011), and the Early Start Denver Model (Dawson et al. 2012). Teachers or other professionals adopting comprehensive program models must commit to being trained and implementing the entire model that might replace a current program or in addition a current program. Because of the differences between the two classes of interventions, and to need to specify a clearly articulated and practical focus for the review, comprehensive program models were not included in this review.

## Previous Literature Reviews of EBPs for Children and Youth with Autism

Before the mid-2000s, the identification of EBPs for children and youth with autism was accomplished through narrative reviews by an individual or set of authors or organizations (e.g., Simpson 2005), which were useful, but did not follow a stringent review process. Many traditional systematic review processes, such as the Cochrane Collaborative (https://www.cochrane.org/) or Project AIM (Sandbank et al. 2020), have only included studies that employed a randomized experimental group design (i.e., randomized control trial or RCT) and have excluded single case experimental design (SCD) studies. By excluding SCD studies, such reviews omit a vital experimental research methodology recognized as a valid scientific approach (What Works Clearinghouse 2020).

It is important to note that SCDs are often excluded in more traditional systematic reviews and meta-analyses, in part because such designs are thought to not be sufficiently scientific or rigorous. For example, on the previously noted Autism Research website (http://www.researchautism.net/autism-interventions/other-aspects-autism-interventions/process-for-evaluating-studies/our-ratings-system), the National Autism Society describes SCD studies as “Grade C” methodology (i.e., contrasted with Grade A and B studies which are group designs). Other reviews have excluded SCD studies entirely (Sandbank et al. 2020). While an individual SCD study provides limited evidence of efficacy, multiple replication studies by different research groups build the strength of evidence. Systematic reviews that minimize or exclude SCD studies ignore the largest body of scientific findings about focused intervention practices. When excluding SCD research, researchers are ignoring the admonition by Sackett et al. (1996): “… if no randomised trial has been carried out for our patient's predicament, we must follow the trail to the next best external evidence and work from there (p. 72).”

Many systematic reviews of interventions for autistic children and youth now appear in the research literature. Such reviews are useful in their focus on individual practices like functional communication training (Gregori et al. 2020), intervention for autistic children/youth of a certain age (Sandbank et al. 2020), or interventions occurring in certain locations such as schools (Martinez et al. 2016), and allow for more in depth review of contextual factors impacting the intervention or outcomes. They do not, however, provide a comprehensive critical summary of evidence across practices, ages, and outcomes. To date, only the National Professional Development Center on ASD (NPDC) and National Standards Project (NSP) have conducted comprehensive reviews of focused intervention practices for children and youth with autism and included both group and SCD studies.

The NSP published their comprehensive review in two phases. In Phase 1, their search process accessed articles from the early years of experimental intervention research for autistic children and youth from 1957 through September 2007 (National Autism Center, 2009). Peer-reviewed journal articles were included if the intervention/treatments were implemented in school, home, community, vocational, clinic settings and included children with autism who did not have significant co-occurring conditions. In Phase 2, the NSP investigators followed the same process as occurred in Phase 1 (National Autism Center, 2015) adding articles published from 2007 to 2012. Their analyses generated 14 practices for children and youth with autism that met their criteria for evidence-based.

The NPDC investigators also conducted two iterations of reviews of the intervention literature. The first review included articles published over the 10-year period from 1997 to 2007 (Odom et al. 2010a, b) and used the research design quality indicator criteria established by the CEC-Division for Research (Gersten et al. 2005; Horner et al. 2005) to evaluate articles for inclusion or exclusion from the review. In the second review, the NPDC team (Wong et al. 2014, 2015) used a more comprehensive search strategy, extended the coverage of the literature to include 22 years of studies (1990–2011), revised their methodological review criteria and process to include current criteria established by What Works Clearinghouse. Using a standard review protocol, they trained a national panel of 159 reviewers to evaluate the methodological quality of the journal articles. From the articles meeting quality criteria, the NPDC team identified 27 EBPs in the second review.

The purpose of this study, now being conducted by the National Clearinghouse for Autism Evidence and Practice (NCAEP), was to update the Wong et al. (2015) review, incorporating autism intervention literature from 2012 to the end of 2017. The questions addressed by this review were: What focused intervention practices are evidence-based? What outcomes areas did evidence-based focused intervention practices address? What are the characteristics of the research designs, participants, and intervention implementation?

## Method

As noted, the previous review (Wong et al. 2015) included articles from 1990 to 2011. In the current review, the methods from the previous review were followed as closely as possible to access and incorporate acceptable articles published from 2012 to 2017. The two groups of articles were then combined for the re-analysis that resulted in the identification of evidence-based practices. The systematic review included five phases: search, screening, quality appraisal, data extraction and synthesis, following the PICO conceptual framework originated by Richardson et al. (1995) and followed by the Cochrane Collaboration. The methods are described in the subsequent sections and more detail about each step of the process can be found in the freely available report on the NCAEP website (https://ncaep.fpg.unc.edu/).

### Search Process

The NCAEP research team and a research librarian from the University of North Carolina at Chapel Hill developed and refined the literature search plan. The databases utilized in the search were: Academic Search Premier, Cumulative Index to Nursing and Allied Health Literature (CINAHL), Excerpta Medica Database (EMBASE), Educational Resource Information Center (ERIC), PsycINFO, Social Work Abstracts, PubMed, Thomson Reuters (ISI) Web of Science, and Sociological Abstracts. Search terms were intentionally broad to be as inclusive as possible and included terms related to diagnosis (autism OR autistic OR Asperger OR ASD OR ASC [autism spectrum condition] OR pervasive developmental disorder OR PDD/PDD-NOS) and practice (intervention OR treatment OR practice OR strategy OR therapy OR program OR procedure OR approach OR methods OR education OR curriculum). The only filters used were peer-reviewed, language (English) and publication date (2012–2017). Deduplication was used to eliminate duplicate articles from the initial search prior to screening.

### Screening Process

Prior to the title/abstract and full-text phases of the screening process, NCAEP team members participated in trainings to review the inclusion and exclusion criteria (see subsequent sections) and screening procedures. For the title/abstract screening, the NCAEP team reviewed the title and abstract of the article and indicated if the article should be excluded or further reviewed in a full-text screening. Following the title/abstract screening, the team gathered the full-text version of all articles that were not excluded. During the full-text screening, team members indicated if an article should be included or excluded, and for excluded articles indicated a reason for exclusion. Both steps were completed internally by single reviewers.

#### Inclusion/Exclusion Criteria for Studies in the Review

Articles included in this review were published in peer-reviewed, English language journals between 1990 and 2017. The study inclusion criteria are described in the subsequent sections and summarized in Table 1.

**Table 1:** Inclusion and exclusion criteria

Category	Inclusion	Exclusion
Literature	Article published (or online prepublication) in peer-reviewed journal	Grey literature such as dissertations, conference presentations or proceedings
Language	Article published in English	Article published in non-English journal
Intervention	Intervention was focused intervention practice Intervention was behavioral, developmental, academic and/or vocational	Intervention was comprehensive treatment program Intervention was medical or psychopharmacological
Outcomes	Outcomes were behavioral, developmental, academic, mental health or vocational for autistic children and youth	Outcomes were physical health, neuroimaging, or EEG Only outcomes for family or caregivers reported
Study Design	Article examined efficacy of intervention with group or single case design	Article primarily descriptive or correlational Article tested moderation of effects on previously published or nonsignificant main effects
Population/ Participants	Some participants identified as autistic Some participants between birth and 22 years of age	Outcomes for participants with autism/in specified age range were not presented separately

##### Population/Participants

A study had to have participants whose ages were between birth and 22 years of age and were identified as having autism spectrum disorder (ASD), autism, Asperger syndrome, pervasive developmental disorder, pervasive developmental disorder-not otherwise specified, or high-functioning autism. Studies varied on the description of the autism diagnosis- some had ADOS or ADIR data, others reported clinical diagnoses by psychiatrists or physicians, while others used diagnoses provided by public school evaluations. Participants with autism who also had co-occurring conditions (e.g., intellectual disability, genetic syndrome, mental health conditions) were included in this review. Studies with participants identified as “at risk for autism” were not included in the review.

##### Interventions

The focused intervention practices examined in a study had to be behavioral, developmental, and/or educational in nature. Studies in which the independent variables were only medications or nutritional supplements/special diets (e.g., melatonin, gluten-casein free, vitamins) were excluded from the review. In addition, only interventions that could be practically implemented in typical educational, clinical, home, or community settings were included. As such, intervention practices requiring highly specialized materials, equipment, or locations unlikely to be available in most educational, clinic, community, or home settings were excluded (e.g., dolphin therapy, hippotherapy, hyperbaric chambers). Interventions requiring the supervision of trained medical personnel were excluded (e.g., chelation, neurofeedback, or acupuncture/acupressure).

##### Outcomes

Studies had to generate behavioral, developmental, academic, vocational, or mental health outcomes (i.e., dependent variables). Outcome data could be discrete behaviors (e.g., social initiations, stereotypies) assessed observationally, ratings of behavior or student performance (e.g., parent/teacher questionnaires), standardized assessments (e.g., nonverbal IQ tests, developmental assessments), and/or informal assessment of student academic performances (e.g., percentage of correct answers for instructional task). Studies only reporting physical health outcomes were excluded. Studies that targeted only caregiver and/or staff outcomes or only examined how those outcomes mediated student outcomes were excluded.

##### Study Designs

Studies included in the review had to employ a group design or SCD to test the efficacy of focused intervention practices. Adequate group designs included randomized control trials, sequential multiple assignment randomized trials, quasi- experimental designs, or regression discontinuity designs that compared an experimental/intervention group receiving the intervention to a control or comparison group that did not receive the intervention or received another intervention (Shadish et al. 2002). SCDs had to demonstrate the functional relationships between the intervention (or independent variable) and the autistic child/youth outcomes (Kazdin 2011). Acceptable SCDs for this review were withdrawal of treatment (ABAB), concurrent multiple baseline, multiple probe, alternating treatment, and changing criterion designs (Horner and Odom 2014), as well as SCDs that included hybrid designs (e.g., an ABAB set of phases in an multiple baseline design). Studies that were solely descriptive, examined only predictors, reviewed existing literature, or were meta-analyses were excluded. In addition, non-concurrent multiple baseline studies and component analyses without a baseline condition were also excluded.

### Quality Appraisals

Protocols for reviewing group design and SCD studies used to determine methodological acceptability were developed in the Wong et al. (2015) review and appear in the supplemental materials for this article. The protocols drew from the methodological quality indicators developed by Gersten and colleagues (2005) for group design and Horner et al. (2005) for SCD, as well as the review guidelines established by the WWC. In addition, the last item on each protocol asked reviewers to make a judgement about whether the study reported positive effects for the intervention. Protocols went through two iterations of pilot testing within the research group and then were reviewed by two national leaders in research methodology and intervention research, with expertise in SCD and group design, respectively for finalization. Only minor updates were made in the current review (e.g., include SMART design as a design option).

#### National Board of Reviewers

To assist in quality appraisals, external reviewers were recruited through professional organizations (e.g., Association for Behavior Analysis International, Council for Exceptional Children), professional contacts, social media, the NCAEP website, and solicitation to reviewers from the previous review. The criteria for qualifying for the subsequent training was that the individual had to have a graduate degree or be a current Ph.D. student, had to have finished coursework in experimental group design and/or single case design research, and had to have had coursework related to and/or experience working with individuals with autism. The reviewers self-identified their methodological expertise and interests as group, SCD, or both. Reviewers completed an online training process described fully in the project report (http://go.unc.edu/Hk72T). Following training, they coded a “master-file” article (i.e., an article in which correct review answers had been established by our team) that employed the respective design. For the review of the master-file study, reviewers had to meet an 80% inter-rater agreement criterion for study elements. If potential reviewers did not meet the criteria, they were allowed to review the training materials and complete the task a second time (i.e., with a different master-file article for the article review).

Two hundred and twenty-one reviewers completed the training and met inter-rater agreement criteria with the master code files; 55% completed requirements for single case design articles (n = 122), 10% completed requirements for group design articles (n = 21), and 35% completed requirements for both design types (n = 78). Most reviewers received their degrees in the area of special education or applied behavior analysis and were faculty, graduate students, or practitioners. Reviewers were acknowledged in the report and BCBA/BCaBA reviewers received continuing education credit if requested.

Ten articles were randomly assigned to each reviewer, with the exception that a check was conducted after assignment to make sure that reviewers had not been assigned an article for which they were an author. They first completed a set of screening questions about the articles (e.g., type of study design) followed by the methodological review items for SCD or group design. If an article met all individual methodological items on the protocol, reviewers noted whether the study had positive effects for autistic participants on at least one outcome variable and listed the variables with positive effects. Last, reviewers described the key features of the study (e.g., participant characteristics) and the intervention procedures. Each article was independently reviewed by two external reviewers. Once both reviews for a given article were complete, the NCAEP team identified any disagreements between the reviewers related to methodological quality and effects. If needed, an NCAEP team member was assigned to complete a third review and make a final determination about quality and/or effects. A third review was required for 42% of the articles, including 27% that were reviewed for quality and effects and 15% that were reviewed for effects only.

##### Inter-rater Agreement

The NCAEP team collected inter-rater agreement on quality appraisals for 1,085 articles. The formula for inter-rater agreement was rating agreements divided by agreements plus disagreements multiplied by 100%. Agreement was calculated for (a) methodological quality review items on the review protocol, (b) summative evaluation of whether a study met or did not meet quality criteria, and (c) evaluation of whether or not studies that met quality criteria had positive effects for autistic participants on at least one outcome variable. Mean inter-rater agreement on the individual items for quality review was 85% (range = 55–97%) for group design articles and 93% (range = 87–97%) for SCD articles, generating a total mean item agreement of 90%. Mean inter-rater agreement for summary decisions about article inclusion was 65% for group design articles and 80% for SCD articles, generating a total agreement of 73%. This agreement percentage was lower because if a single reviewer rated even a single item as negative, it led to exclusion of the article from the study. As noted, when a disagreement between reviewers occurred, a third internal review was conducted by an NCAEP staff member, which led to a consensus decision about inclusion or exclusion of the article. Of the included articles, there was 86% agreement for group design articles and 74% agreement for SCD articles on the presence of positive effects, yielding a total agreement of 80% (i.e., between the respective reviewers who agreed on the previous inclusion decision). When disagreement occurred on the positive effects report, internal NCAEP staff conducted another review just focusing on the decision about positive effects to yield a consensus judgment.

### Data Extraction

The NCAEP Team followed a three-step data extraction process. First, team members compared external reviewers reports of participant characteristics (e.g., diagnosis, age) and outcomes (i.e., dependent variables), and made final determinations for this information. Second, team members thoroughly reviewed each article to identify primary interventions. In this identification step, the reviewer could: assign an article to one or more of the 27 practice categories identified in the previous review (Wong et al. 2014); assign the article to a practice category that had been identified as having some evidence in the previous review; and/or identify a new possible category of practices represented in the article. Third, different team members reviewed each article assigned to a given practice in the previous step to confirm that it fit within the practice description. During this step of data extraction, team members also identified manualized interventions that fit within a practice. Manualized interventions shared procedural features that were similar to other interventions in the category but had unique features that distinguished them as a salient model and had an identifiable title. For example, Social Stories™ is a trademarked intervention by Carol Gray and Garand (1993), that fits within Social Narratives practice description, but is also distinct as a particular type of Social Narrative. Also, during the data extraction phase of the review, our team identified additional articles that were removed for not meeting eligibility requirements and/or quality standards, which may have been missed in the original quality review. These decisions were confirmed by a second team member. Then, the data were compiled for analysis and synthesis.

### Synthesis and Identification of EBPs

When all included articles were assembled into categories, the team made a final determination about whether a practice met the level of evidence necessary to be classified as an EBP using criteria for evidence established by the previous NPDC team. The NPDC’s criteria were drawn from the work of Nathan and Gorman (2007), Rogers and Vismara (2008), Horner and colleagues (2005), and Gersten and colleagues (2005), as well as the earlier work by the APA Division 12 (Chambless and Hollon 1998). Its rationale is based on the necessity of having a sufficient number of empirical demonstrations of efficacy through high quality, peer-reviewed journal articles and replications of those demonstrations by independent research groups.

Different criteria were established for group and single case design evidence. To be identified as evidence-based, a category of practice had to contain (a) two high quality group design studies conducted by two different research groups, (b) five high quality single case design studies conducted by three different research groups and involving a total of 20 participants across studies, or (c) a combination of one high quality group design study and three high quality single case design studies with the combination being conducted by two independent research groups. Independence of research groups was defined as the research being located in different settings and the key constituent members of the authorship of published articles being different from other research groups.

## Results

### Search Results

**Fig. 2:**
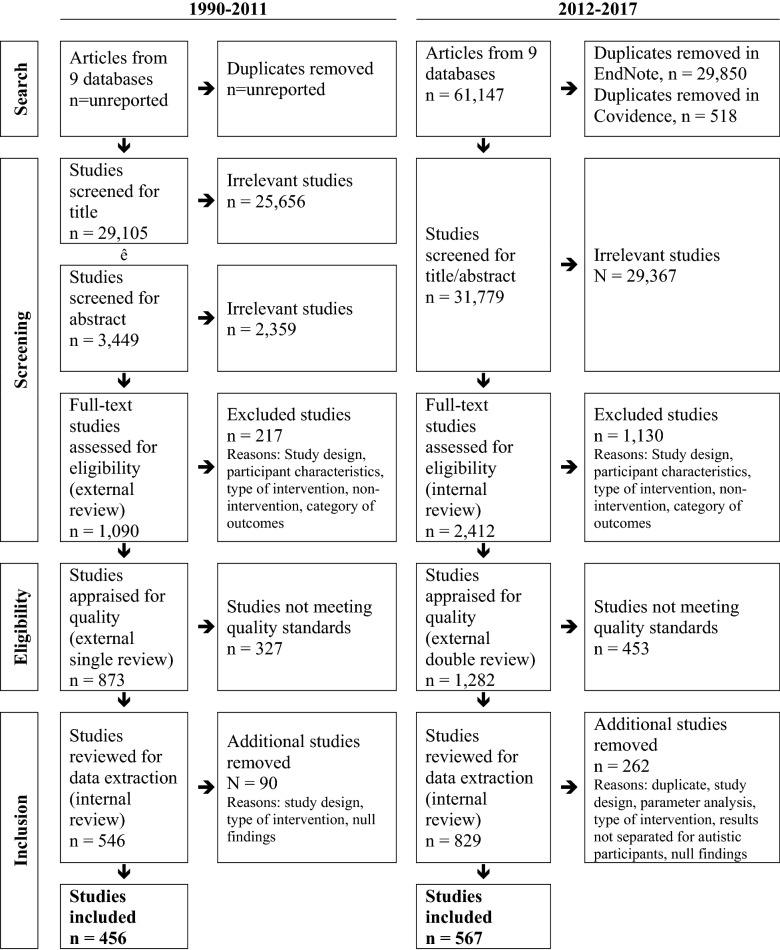
PRISMA flow charts

The search update incorporated results from the nine databases which generated an initial total of 61,147 articles and 31,779 after duplicates were removed. Following the title/abstract and full-text screening, quality appraisal, and a final review during data extraction, 567 articles met the established criteria for evidence and showed positive effects for at least one relevant outcome. Figure 2 is the PRISMA chart that shows the articles excluded and included at each of the steps for both the previous (1990–2011) and current (2012–2017) review periods.

**Fig. 3:**
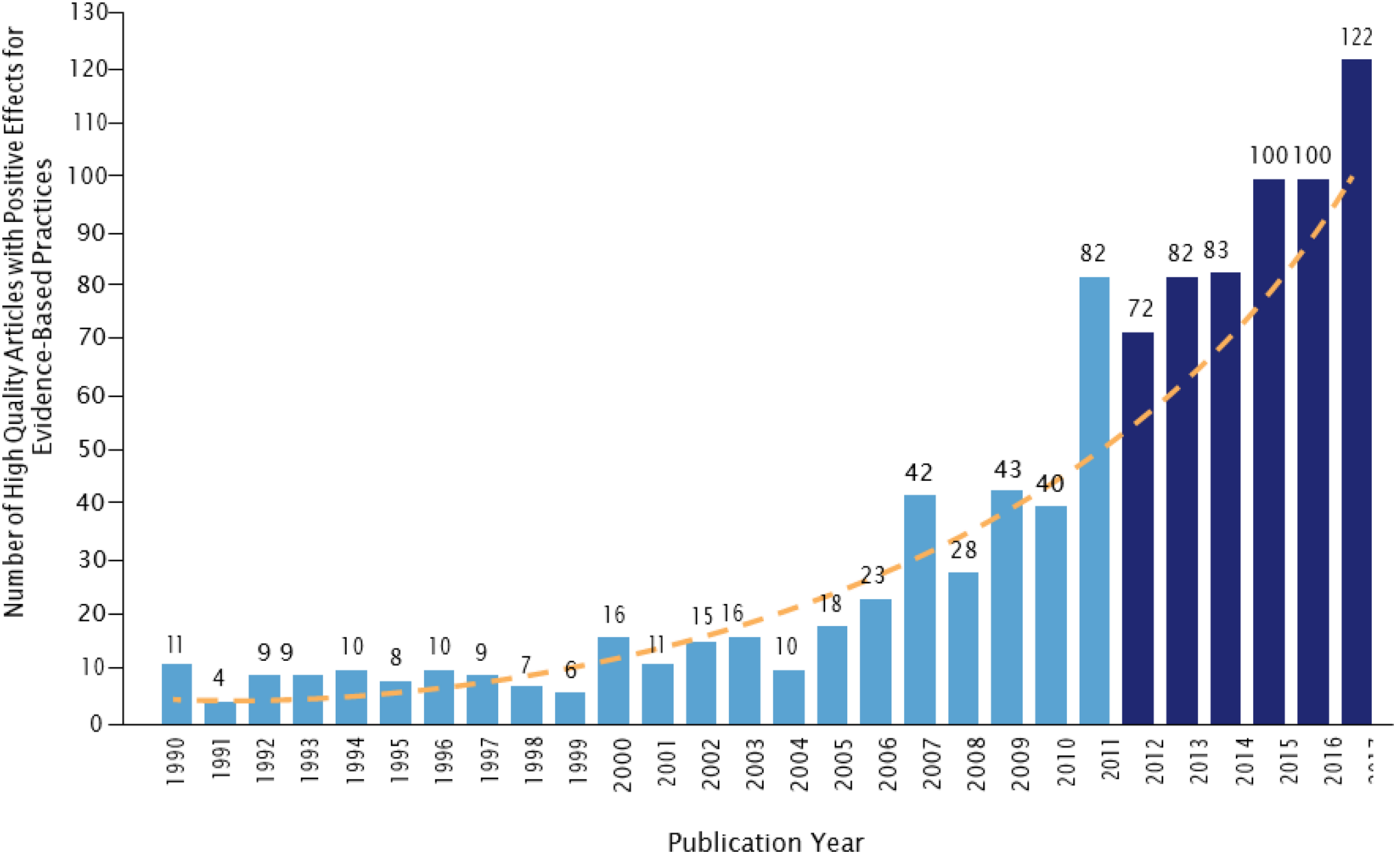
Increase in accepted articles across time

The NCAEP team reviewed the remaining 567 articles with positive effects and determined that 545 of the articles were primary studies (i.e., not secondary data analysis or follow-up analysis of a primary study in the review). These 545 studies were combined with 427 studies from the previous review, yielding a total of 972 acceptable articles. The number of articles by year of publication appears in Fig. 3, demonstrating a clear acceleration in acceptable articles from 1990 to 2017.

### Participant Characteristics

Paricipants were described by diagnosis, age, sex, and race/ethnicity/nationality.

#### Diagnosis and Co-morbid Conditions

Autism was the most frequently reported diagnosis in studies, with 64% of studies indicating at least one participant with autism (a given study could include multiple diagnostic or co-occurring conditions categories). There was, however, a drop in the use of “autism” as a descriptor from the 1990–2011 period (83%) to the 2012–2017 period (48%). There was a large increase in reporting of “ASD” as a diagnosis, moving from 12% (1990–2011) to 55% (2012–2017). The reports of participants with “Asperger” or “High Functioning Autism” (HFA) and “PDD” or “PDD-NOS” were relatively low (10% and 14%) and stayed fairly stable across review periods. Although 55% of the studies failed to report any information on co-occurring conditions, when reported, the most frequent co-occurring condition was intellectual disability (21%).

#### Age

Participants’ ages were classified into six categories and multiple age categories could be selected for each study. The majority of studies were conducted with preschool (43%) and elementary-aged children (57%). When comparing the 1990–2011 and 2012–2017 review periods, there were substantial increases in the percentages of studies conducted with 12–14-year-olds (17% and 27% respectively) and 15–18-year-olds (10% and 17% respectively). The youngest age category, birth-35 months, had a slight increase (6% to 9%) and the oldest age category, 19–22 years, remained stable across review periods at 5%.

#### Gender/Sex

In the previous review, data on gender and sex were not extracted so these data only reflect the 2012–2017 review period. Data on the gender or sex of the participants were reported in 93% of studies. Although “non-binary” and “other” were included as options during the data extraction, no studies reported these categories. In studies that reported the number of participants in the gender or sex categories, 84% of participants were male.

#### Race/Ethnicity/Nationality

Data on race/ethnicity/nationality were also not extracted in the 1990–2011 studies, so these data reflect the 2012–2017 review period. Thirty percent of the studies reported data on race/ethnicity/nationality. For studies that reported numbers of participants by categories, 59% of the participants were White, 10% were Black, 9% were Asian, and 8% were Hispanic/Latino. All other groups had less than 5% representation among participants in studies reporting this information.

### Study Design Types

Across the studies for both review periods, single case design studies made up 83% of the articles and group design made up 17%. The primary group design employed was a randomized control design (14% of total), followed by quasi-experimental design (3%), and one sequential multiple assignment randomized trials design. For single case designs, the multiple baseline (31%) and multiple probe designs were the most frequently used (14%) followed by withdrawal of treatment (12%). One notable change is that 23% were group designs for the 2012–2017 set of studies, compared to only 9% of the studies in the previous review period.

### Implementation Characteristics

Data on implementation characteristics were only extracted from the 2012–2017 review period. Research staff implemented (i.e., directly provided) interventions in 52% of the studies and were coaches in 10% of studies. Educators and related service providers were each identified as implementers in 20% of studies, and parents were noted as implementers in 10% of studies. In 48% of studies the intervention took place in educational settings. The other intervention locations were university clinic/research lab settings (20% of studies), home settings (18%), and community clinic settings (13%). On occasion, studies took place in more than one setting so multiple locations could be selected for a single study. Seventy-nine percent of the studies were conducted in individual sessions (i.e., one-on- one), and 14% were conducted in small group settings with 3–6 total participants. All other group sizes occurred in less than 6% of the studies.

### Evidence-Based Practices

Twenty-eight practices met the criteria for being evidence-based. The 28 EBPs, their abbreviated definitions, the number of articles from each review period, and the number of articles from each study type (i.e., SCD, group) that contributed to the evidence base are included in Table 2. The specific studies supporting the practice are listed in the original report (Steinbrenner et al. 2020). There are five new EBP categories in this review: Behavior Momentum Intervention, Direct Instruction, Music-Mediated Intervention, Sensory Integration (i.e., explicitly the model developed by Ayres 2005), and Augmentative and Alternative Communication (AAC; includes practices previously in other categories).

**Table 2:** Evidence-based practices, definitions and number of articles across review periods

Evidence-based practice	Definition	Empirical support				
		Year			Study design	
		1990–2011 (*n*)	2012–2017 (*n*)	1990–2017 (*n*)	Group	SCD
Antecedent-based interventions (ABI)	Arrangement of events or circumstances that precede an activity or demand in order to increase the occurrence of a behavior or lead to the reduction of the challenging/interfering behaviors	29	20	49	2	47
Augmentative and alternative communication (AAC)	Interventions using and/or teaching the use of a system of communication that is not verbal/vocal which can be aided (e.g., device, communication book) or unaided (e.g., sign language)	9	35	44	5	39
Behavioral momentum intervention (BMI)	The organization of behavior expectations in a sequence in which low probability, or more difficult, responses are embedded in a series of high probability, or less effortful, responses to increase persistence and the occurrence of the low probability responses	8	4	12	0	12
Cognitive behavioral/instructional strategies (CBIS)	Instruction on management or control of cognitive processes that lead to changes in behavioral, social, or academic behavior	7	43	50	34	16
Differential reinforcement of alternative, incompatible, or other behavior (DR)	A systematic process that increases desirable behavior or the absence of an undesirable behavior by providing positive consequences for demonstration/non-demonstration of such behavior. These consequences may be provided when the learner is: (a) engaging in a specific desired behavior other than the undesirable behavior (DRA), (b) engaging in a behavior that is physically impossible to do while exhibiting the undesirable behavior (DRI), or (c) not engaging in the undesirable behavior (DRO)	27	31	58	0	58
Direct instruction (DI)	A systematic approach to teaching using a sequenced instructional package with scripted protocols or lessons. It emphasizes teacher and student dialogue through choral and independent student responses and employs systematic and explicit error corrections to promote mastery and generalization	2	6	8	1	7
Discrete trial training (DTT)	Instructional approach with massed or repeated trials with each trial consisting of the teacher’s instruction/presentation, the child’s response, a carefully planned consequence, and a pause prior to presenting the next instruction	16	22	38	2	36
Exercise and movement (EXM)	Interventions that use physical exertion, specific motor skills/ techniques, or mindful movement to target a variety of skills and behaviors	6	11	17	11	6
Extinction (EXT)	The removal of reinforcing consequences of a challenging behavior in order to reduce the future occurrence of that behavior	13	12	25	0	25
Functional behavioral assessment (FBA)	A systematic way of determining the underlying function or purpose of a behavior so that an effective intervention plan can be developed	11	10	21	0	21
Functional communication training (FCT)	A set of practices that replace a challenging behavior that has a communication function with more appropriate and effective communication behaviors or skills	12	19	31	0	31
Modeling (MD)	Demonstration of a desired target behavior that results in use of the behavior by the learner and that leads to the acquisition of the target behavior	10	18	28	2	26
Music-mediated intervention (MMI)	Intervention that incorporates songs, melodic intonation, and/or rhythm to support learning or performance of skills/behaviors. It includes music therapy, as well as other interventions that incorporate music to address target skills	3	4	7	3	4
Naturalistic intervention (NI)	A collection of techniques and strategies that are embedded in typical activities and/or routines in which the learner participates to naturally promote, support, and encourage target skills/behaviors	26	49	75	37	38
Parent-implemented intervention (PII)	Parent delivery of an intervention to their child that promotes their social communication or other skills or decreases their challenging behavior	13	42	55	28	27
Peer-based instruction and intervention (PBII)	Intervention in which peers directly promote autistic children’s social interactions and/or other individual learning goals, or the teacher/ other adult organizes the social context (e.g. play groups, social network groups, recess) and when necessary provides support (e.g., prompts, reinforcement) to the autistic children and their peer to engage in social interactions	19	25	44	8	36
Prompting (PP)	Verbal, gestural, or physical assistance given to learners to support them in acquiring or engaging in a targeted behavior or skill	55	85	140	7	133
Reinforcement (R)	The application of a consequence following a learner’s use of a response or skills that increases the likelihood that the learner will use the response/skills in the future	53	53	106	2	104
Response interruption/redirection (RIR)	The introduction of a prompt, comment, or other distractors when an interfering behavior is occurring that is designed to divert the learner’s attention away from the interfering behavior and result in its reduction	13	16	29	0	29
Self-management (SM)	Instruction focusing on learners discriminating between appropriate and inappropriate behaviors, accurately monitoring and recording their own behaviors, and rewarding themselves for behaving appropriately	14	12	26	1	25
Sensory integration (SI)	As originated by A. Jean Ayres interventions that target a person’s ability to integrate sensory information (visual, auditory, tactile, proprioceptive, and vestibular) from their body and environment in order to respond using organized and adaptive behavior	1	2	3	3	0
Social narratives (SN)	Interventions that describe social situations in order to highlight relevant features of a target behavior or skill and offer examples of appropriate responding	15	6	21	1	20
Social skills training (SST)	Interventions that describe social situations in order to highlight relevant features of a target behavior or skill and offer examples of appropriate responding	18	56	74	40	34
Task analysis (TA)	A process in which an activity or behavior is divided into small, manageable steps in order to assess and teach the skill. Other practices, such as reinforcement, video modeling, or time delay, are often used to facilitate acquisition of the smaller steps	9	4	13	0	13
Technology-aided instruction and intervention (TAII)	Instruction or intervention in which technology is the central feature and the technology is specifically designed or employed to support the learning or performance of a behavior or skill for the learner	10	30	40	23	17
Time delay (TD)	A practice used to systematically fade the use of prompts during instructional activities by using a brief delay between the initial instruction and any additional instructions or prompts	16	15	31	0	31
Video modeling (VM)	A video-recorded demonstration of the targeted behavior or skill shown to the learner to assist learning in or engaging in a desired behavior or skill	35	62	97	2	95
Visual supports (VS)	A visual display that supports the learner engaging in a desired behavior or skills independent of additional prompts	34	31	65	3	62

The inclusion of the new literature from 2012 to 2017 led to some recategorization and reconceptualization of EBPs from the previous review. PECS® was merged with AAC, Pivotal Response Training (PRT) was merged into Naturalistic Interventions, Scripting was merged into Visual Supports. Also, Peer-Mediated Intervention and Instruction and Structured Play Groups were merged into the new category of Peer-based Intervention and Instruction. Exercise was broadened to Exercise and Movement, and Cognitive Behavioral Intervention was broadened to Cognitive Behavioral/Instructional Strategies.

### Manualized Interventions Meeting Criteria for EBPs

Emerging from the current review were interventions that clearly fit the EBP categorical definitions but had themselves enough evidence to be classified as an EBP. The NCAEP team identified these practices as Manualized Interventions Meeting Criteria (MIMCs) and grouped them within established EBP categories. The rationale for this classification was to provide conceptual clarity of the EBP organization but also to highlight the particular approach. In addition to having sufficient evidence, MIMCs had to have clearly established manualized procedures or software. In total, there were 10 MIMCs classified within six of the EBP categories. These MIMCs appear in Table 3. More detail about the reclassification process may be found in the full report (Steinbrenner et al. 2020).

**Table 3:** Manualized interventions meeting criteria (MIMCs)

MIMC	Associated EBP	Relevant references
Picture exchange communication system® (PECS)	Augmentative and alternative communication	Frost and Bondy (2002)
JASPER	Naturalistic intervention	Kasari et al. (2014)
Milieu Teaching	Naturalistic intervention	Kaiser and Roberts (2013)
Pivotal Response Training	Naturalistic intervention	Koegel and Koegel (2006)
Project ImPACT	Parent-implemented intervention	Ingersoll and Dvortcsak (2019)
Stepping stones triple P	Parent-implemented intervention	Turner et al. (2010)
Social stories™	Social narratives	Gray (2000)
PEERS®	Social skills training	Laugeson and Frankel (2010)
FaceSay ®	Technology-aided instruction and intervention	Hopkins et al. (2011)
Mindreading	Technology-aided instruction and intervention	Golan and Baron-Cohen (2006)

### Outcomes

The child/youth outcomes addressed by studies in this review appear in Table 4, along with the number of studies supporting each reported in the earlier and current review. Communication, social, and challenging/interfering behavior were outcomes addressed most often. Outcomes with increased number of studies from the previous to current review were academic, mental health, and vocational. Self-determination was added as an outcome. Outcomes for which there were fewer articles in the most recent review update (2012–2017) as compared to the previous review were challenging behavior, joint attention, play, and school readiness.

**Table 4:** Outcomes identified across review periods

Domain/instructional outcome	Definitions	1990–2011 (*n*)	2012–2017 (*n*)	1990–2017 (*n*)
Academic/pre-academic	Outcomes broadly related to performance on tasks typically taught and used in school settings	55	96	151
Adaptive/self-help	Outcomes related to independent living skills and personal care skills	52	53	105
Challenging/interfering behavior	Outcomes related to decreasing or eliminating behaviors that interfere with the individual’s ability to learn	147	121	268
Cognitive	Outcomes related to performance on measures of intelligence, executive function, problem solving, information processing, reasoning, theory of mind, memory, creativity, or attention	15	22	37
Communication	Outcomes related to ability to express wants, needs, choices, feelings, or ideas	173	159	332
Joint attention	Outcomes related to behaviors needed for sharing interests and/or experiences	36	27	63
Mental health	Outcomes related to emotional well-being	1	16	17
Motor	Outcomes related to movement or motion, including both fine and gross motor skills, or related to sensory system/sensory functioning	17	16	33
Play	Outcomes related to the use of toys or leisure materials	73	50	123
Self-determination	Outcomes related to self-directed actions in setting and achieving goals or making decisions and problem-solving	0	2	2
School readiness	Outcomes related to task performance versus task content or curriculum area (e.g., on task behavior, engagement)	63	46	109
Social	Outcomes related to skills needed to interact with others	152	150	302
Vocational	Outcomes related to employment or employment preparation or relate to technical skills required for a specific job	11	20	31

**Fig. 4:**
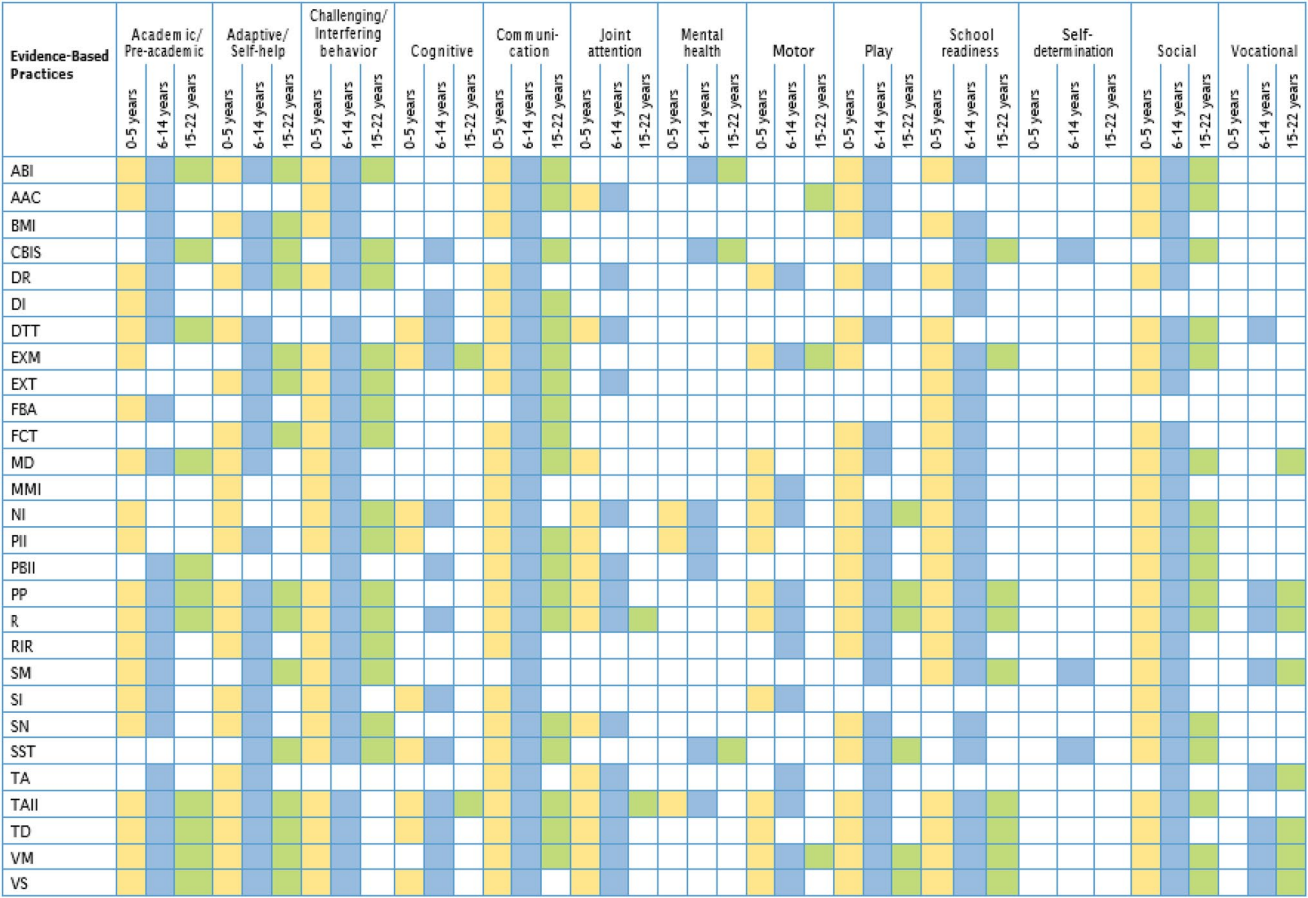
Matrix of evidence-based practices, outcomes, and age categories

A matrix that displays the outcomes identified for each EBP, also sorted by age group within the EBP can be found in Fig. 4. The filled cells indicate that at least one study generated the indicated outcome for an age group (from the column) for a specific intervention (from the row). Most of the EBPs have at least some evidence of impact across a wide variety of ages (three or more age groups). In general, EBPs tend to address a wide variety of outcome categories, ranging from four to 11 outcomes. Notably, 23 EBPs have been shown to impact seven or more outcome categories and 16 EBPs have been shown to impact nine or more.

## Discussion

The current report updates and extends the work on evidence-based, focused intervention practices begun with an initial review of the literature from 1997 to 2007 (Odom et al. 2010a, b) and extended through a second report that covered the literature from 1990 to 2011 (Wong et al. 2015); extending this systematic review through 2017 added 567 articles to the review. As the intervention literature has provided more empirical information and as practices have evolved, some of the classifications required reconceptualization and revision of previous definitions. In an active research area, knowledge does not stand still, and in fact identification of EBPs should be dynamic, reflecting the growth of knowledge across time (Biglan and Ogden 2019).

The five new practices in this review are Augmentative and Alternative Communication (AAC), Behavior Momentum Intervention, Direct Instruction, Music-Mediated Intervention, and Sensory Integration. It is important to note that Sensory Integration refers explicitly to the classical sensory integration model developed by Jean Ayres (2005) and not to a variety of interventions that address sensory issues but have been found to be unsupported (Case-Smith et al. 2015; Watling and Hauer 2015). Also, several EBP categories from previous reviews were reclassified into other EBP categories (i.e., Scripting into Visual Supports, Peer-Mediated Intervention and Instruction and Structured Play Groups into Peer-Based Intervention and Instruction).

The growth of the published empirical literature led to a new classification of interventions noted above as MIMC. These interventions were developed by individual research groups and had sufficient evidence to meet the criteria as an EBP. However, the procedural features overlapped directly with established EBP categories. As such, MIMC could be considered “EBPs within broader EBP categories.” Ten interventions met the criteria for MIMC and have sufficient evidence to meet the EBP criteria. In addition, these interventions have clearly established manualized procedures or software, often including training protocols, which may better facilitate their uptake and implementation (Kasari and Smith 2013). Two interventions, PECS® (Frost and Bondy 2002) and PRT (Koegel and Koegel 2006; Stahmer et al. 2011), previously identified as EBP categories were reclassified under AAC and Naturalistic Intervention, respectively. The MIMC classification does not convey in any way that the interventions are less strong or efficacious than previously indicated, because they both have an extensive set of research that supports their efficacy. Also, some MIMCs have features that cross EBP categories. For example, Project ImPACT (Ingersoll and Dvortcsak 2019) was classified within the Parent-Implemented EBP but it also shares some characteristics with Naturalistic Intervention.

Because this review included articles for two time periods, it was possible to examine trends across time. In our analysis, SCD remained the primary methodology employed, constituting 85% of the acceptable studies. Multiple baseline and multiple probe designs were more frequently used in the more current set of articles as compared to the earlier review, perhaps because they do not require a treatment to be withdrawn in order to demonstrate an experimental effect of the intervention (i.e., as is necessary in ABAB designs). Although group designs were only 17% of the studies included overall, there was a dramatic increase in the number of RCT studies included between the review periods, which may reflect greater access to the number of participants with autism needed to establish power for RCT analyses and/or priorities of funding agencies.

Information on the intervention setting, implementer, and group size was available from the 2012–2017 review period. Commenting on the earlier research literature, Parsons and Kasari (2013) lamented the fact that most intervention research was not occurring in the educational settings where many children and youth with autism spend a great part of their life. In the current review, nearly 50% of the research was conducted in education settings, the largest of any setting reported. While an important step in the right direction, the majority of the research was still being conducted in individual sessions by research staff members. Certainly, directions for the future would be to examine more often the efficacy of interventions when implemented in “authentic” educational settings by practitioners such as teachers, speech language pathologists, psychologists, and other service providers.

Outcomes for intervention participants shifted somewhat from the 1990–2011 to the 2012–2017 review period. As noted, communication, social, and behavior outcomes occurred most frequently across both review periods, as would be expected given that these are the challenges that define autism. There were notable increases in studies that targeted academic skills, vocational skills, and mental health, although the number of studies addressing these outcomes remains low relative to the number of studies. These outcomes are important, especially for adolescents and young adults with autism, and their low frequency document needed areas for research in the future.

For this review, outcomes were coded by domain and individual dependent variables were not coded. For focused intervention practices, researchers tend to address individual skills or behaviors. Also, for SCD studies, researchers employ dependent measures that can be used repeatedly across time to monitor changes in student performance (Kazdin 2011). A safe assumption is that for SCD studies, the outcome assessments employed observational or rating scale data. For group design studies, these methodologies may also be used, but researchers also would have more frequently employed standardized, norm-referenced measures. Describing specific assessment methodology of EBP studies would certainly be a feature of future research.

A number of demographic findings have implications for future research as well. As in the earlier review, the majority of the 2012–2017 studies were conducted with preschool and elementary-aged children with autism, indicating an important need for increased research with infants/toddlers, adolescents, and young adults with autism. Also, although it fits the overall demographic of autism, most studies were conducted with male participants, and information about differential effects for female participants with autism continues to be under-examined. Finally, there are gaps related to the reporting, inclusion, and analysis of participants with co-occurring conditions, which should be addressed in future studies.

In the 2012–2017 review period, information was collected about whether researchers reported the race/ethnicity/nationality of autistic participants, which did not happen in the previous review. However, West and colleagues (2016) recoded the earlier set of articles to retrieve those data, which can serve as a point of comparison. West et al. found that only 17.9% of the articles from 1990 to 2011 reported race/ethnicity, while in the 2012–2017 review period, 30% of the reviewed articles reported these data. In both sets of studies, Black and Hispanic/Latino were the most frequent nonwhite racial/ethnic categories reported. The number of participants from nonwhite racial and ethnic groups in the subset of studies that reported these data was strikingly low compared to what would be expected based on community demographics. For example, only 8% of research participants were Hispanic/Latino, while in the United States alone, 26% of the school age population identifies as Hispanic (U.S. Department of Education 2017). Also, differential treatment outcomes related to race/ethnicity/nationality were not examined, which is similar to findings by Pierce et al. (2014) in an analysis of studies published between 2000 and 2010. Last, socioeconomic class (SES) of participants is rarely described for autistic participants in research studies, so the possibility of determining how SES affects treatment outcomes is largely not possible.

### Limitations

Several limitations exist for this review. As noted, the review included only studies published from 1990 to 2017. Two limitations exist regarding this timeframe. First, studies that occurred before 1990 were not included, although one might expect early (i.e., pre-1990) studies of important and effective practices to have been replicated in publications over subsequent years. Second, because of the time required to conduct a review of a very large database and involve a national self-selected set of reviewers, there was a lag between the end date for a literature review (i.e., 2017) and the date which the review is published. Certainly, studies have been published in the interim that could have impacted the EBP classifications.

Regarding the methodology of the review, this was clearly a systematic review of the literature and not a meta-analysis. So, the magnitude of effect size was not examined. Also, the review only contained peer-reviewed journal articles and not grey literature, as sometimes occurs in meta-analyses (McAuley et al. 2000). In addition, studies with null findings were not included. In fact, experimental studies are rarely set up to prospectively test for null findings, although there are methodological procedures for addressing such a question (Greene et al. 2007). Research studies with a hypothesis of treatment condition differences that instead “prove the null hypothesis” run the greater risk of Type II error. Last, although methodological quality indicators were drawn from recognized authoritative sources in the field, it is possible that a more detailed analysis of methodology than was practical for this review could have influenced the studies included in this review.

### Implications and Conclusions

The current review, as noted previously, is an important link in the research to practice process. When translated into useable and accessible information for practitioners (Sam et al. 2020a, b) and supported through professional development and implementation science (Sam et al. 2020a, b), these practices can be essential components of effective programs for autistic children and youth. That is, practitioners may match EBPs to specific learning goals for autistic children and youth (Cox and Sam n.d.) analogous to the way medical practitioners match specific treatments to the health needs and characteristics of their patients in personalized medicine. By assembling multiple EBPs to address specific learning needs, practitioners can build a technical eclectic program for children and youth with autism (Lazarus and Beutler 1993). In such an approach, practitioners establish programs with strong program quality as a foundation, develop individualized and clearly articulated goals for children/youth, select and implement practices that may have different theoretical bases but also have demonstrated efficacy (Odom et al. 2012). In a recent study, Sam et al. (2020a, b) employed such a program in 59 elementary schools and found significantly positive effects for program quality, teachers use of EBP with fidelity, and autistic children goal attainment.

In conclusion, the current study provided an updated review of the empirical evidence supporting focused intervention practices. In this review, nearly 1,000 studies were identified, with over half being published between 2012 and 2017. The updated review led to a revised set of 28 primary EBPs and also 10 interventions that classified as MIMCs. Examination of trends from the earlier review and current update suggest that SCD studies continue to be the modal form of research although RCT are being used more often. This analysis suggests that important directions for future research include intervention effects related to race, ethnicity, and gender as well as increased research for both infants/toddlers and adolescents/young adults with autism.

## Supplementary Information

Below is the link to the electronic supplementary material.

Supplementary file1 (PDF 160 KB)

## Footnotes

1 In this document, we will use a mixture of terminology when referring to autism and persons identified as autistic. A common form of description has been called “person-first”, in which the person (e.g., child) appears before the condition (e.g., autism), such as child with autism. Many autistic self-advocates and advocacy groups now prefer an identify-first form, such as autistic child (Brown 2011; Kenny et al. 2016). In addition, autistic advocates have spoken about the desirability of using the term “autism” rather than autism spectrum disorder (Brown, 2011). At the time of this writing, terminological issues have not been settled. To honor the advocates and professionals in the field, as well as other groups of individuals with disabilities who prefer the person-first term, we will be mixing terminology throughout the manuscript, using both person-first and identity-first terminology with the primary descriptor being autism or autistic.
