# Correction to: Evidence-Based Practices for Children, Youth, and Young Adults with Autism: Third Generation

**DOI:** 10.1007/s10803-022-05438-w

**Published:** 2022-01-20

**Authors:** Kara Hume, Jessica R. Steinbrenner, Samuel L. Odom, Kristi L. Morin, Sallie W. Nowell, Brianne Tomaszewski, Susan Szendrey, Nancy S. McIntyre, Serife Yücesoy-Özkan, Melissa N. Savage

**Affiliations:** 1 School of Education, The University of North Carolina at Chapel Hill, CB 3500 Peabody Hall, Chapel Hill, NC 27599 USA; 2 Frank Porter Graham Child Development Institute, The University of North Carolina at Chapel Hill, Campus Box 8040, Chapel Hill, NC 27599-8040 USA; 3 Present Address: College of Education, Lehigh University, Iacocca Hall, 111 Research Drive, Bethlehem, PA 18015 USA; 4 Department of Allied Health Sciences, The University of North Carolina at Chapel Hill, Bondurant Hall, Campus Box 7120, Chapel Hill, NC 27599-7120 USA; 5 Present Address: College of Health Professionals and Sciences, University of Central Florida, 12805 Pegasus Drive, Orlando, FL 32816 USA; 6 Present Address: Department of Special Education, Anadolu Üniversitesi, Eğitim Fakültesi, Özel Eğitim Bölümü, Tepebaşı, Eskisehir, 26470 Turkey; 7 Present Address: College of Education, University of North Texas, 1300 W. Highland St., Denton, TX 76201 USA

## Correction to: Journal of Autism and Developmental Disorders (2021) 51:4013–4032 10.1007/s10803-020-04844-2

Please note the following corrections to this article:

The article references Sensory Integration® (SI) four times in the text and in Table 2 and Fig. 4. To clarify the practice for which our review found evidence, these should instead read “Ayres Sensory Integration® (ASI®)”. The corrected terminology can be found in all of our web-based materials: https://ncaep.fpg.unc.edu/research-resources

