# Supplementary material for: Evidence-Based Practices for Children, Youth, and Young Adults with Autism: Third Generation Review
Source: J Autism Dev Disord. 2021 Jan 15;51(11):4013–32. doi: 10.1007/s10803-020-04844-2 (PMC8510990; doi:10.1007/s10803-020-04844-2)
Supplement: Supplementary file 1 — Supplementary file1 (PDF 160 KB) [file 10803_2020_4844_MOESM1_ESM.pdf]

Supplementary Materials

Group Design Quality Appraisal Form

Question	Yes	No	Not Reported
Does the study have experimental and control/comparison groups?			
Were appropriate procedures used to increase the likelihood that relevant characteristics of participants in the sample were comparable across conditions? (To meet this standard, one of the following criteria must be met a) participants were randomly assigned across study conditions, b) participants were matched on key demographic variables, OR c) researchers statistically controlled for effects of differing key variables to ensure equivalence of groups.			
Were outcomes for capturing the intervention's effect measured at appropriate times (at least pre- and post-test)?			
Was there evidence for adequate reliability and validity for the key outcome measures? And/or when relevant, was inter-observer reliability assessed and reported to be at an acceptable level?			
Was the intervention described and specified clearly enough that it could be replicated by another interventionist?			
Was the control/comparison condition(s) described?			
Were the data analysis techniques appropriately linked to key research questions and hypotheses?			
Attrition was not a significant threat to internal validity.			
Was the measure of effect attributed to the intervention? (no obvious unaccounted confounding factors)			
Does the research report statistically significant positive effects of the practice for individuals with ASD for at least one outcome variable?			

Single Case Design Quality Appraisal Form

Question	Yes	No	Not Reported
Does the dependent variable align with the research question or purpose of the study?			
Was the dependent variable clearly defined such that another person could identify an occurrence or nonoccurrence of the response?			
Does the measurement system align with the dependent variable and produce a quantifiable index?			
Did a secondary observer collect data on the dependent variable for at least 20% of the sessions across conditions?			
Was mean interobserver agreement (IOA) 80% or greater OR kappa of .60 or greater?			
Is the independent variable described with enough information to allow for a clear understanding about the critical differences between the baseline and intervention conditions, or were references to other published material used if description does not allow for a clear understanding?			
Was the baseline described in a manner that allows for a clear understanding of the differences between the baseline and intervention conditions?<br>*Can select not reported for ATDs only			
Are the results displayed in a graphic format showing repeated measures for a single case (e.g., behavior, participant, group) across time?			
Do the results demonstrate changes in the dependent variable when the independent variable is manipulated by the experimenter at three different points in time or across three phase repetitions?<br>*For ATD, must be at least 4 repetitions of alternating sequence<br>**Changing criterion- baseline plus three intervention phases			
